# Sonomyography accurately captures joint kinematics during volitional and electrically stimulated motion in healthy adults and an individual with cerebral palsy

**DOI:** 10.1186/s12984-025-01784-9

**Published:** 2025-12-11

**Authors:** Shriniwas Patwardhan, Noah Rubin, Katharine E. Alter, Diane L. Damiano, Thomas C. Bulea

**Affiliations:** Rehabilitation Medicine Department, National Institutes of Health Clinical Center, 9000 Rockville Pike, Bethesda, MD 20892 USA

**Keywords:** Ultrasound imaging, Sonomyography, Multimodal sensing, Cerebral palsy

## Abstract

**Background:**

Despite significant advances in biosignal extraction techniques for studying neuromotor disorders, there remains an unmet need for a method that effectively links muscle structure and dynamics to muscle activation. Addressing this gap could improve the quantification of neuromuscular impairments and pave the way for precision rehabilitation. In this study, we demonstrate the proof of concept of recording multimodal signals from the brain, muscles, and resulting limb kinematics. We also explore the use of ultrasound imaging to extract limb kinematics.

**Methods:**

We collected data from three healthy volunteers and one individual with cerebral palsy during single degree-of-freedom ankle and wrist movements. Participants performed range of motion (ROM) tasks at approximately 1-second intervals, either volitionally or through functional electrical stimulation. We simultaneously recorded electroencephalography, surface electromyography (EMG), continuous ultrasound imaging, and motion capture data. Joint kinematics were computed from ultrasound imaging using a technique called sonomyography (SMG), and we evaluated the technical feasibility of estimating joint kinematics from both sonomyography and surface EMG signals.

**Results:**

The technical feasibility study evaluated joint angle prediction using EMG and SMG under volitional (FES-OFF) and electrically stimulated (FES-ON) conditions. Root mean squared error (RMSE) between predicted and measured joint angles was computed for multiple methods of extracting kinematics from EMG and SMG. EMG-based RMSE ranged from 0.34 to 0.57 (FES-OFF) and 0.43–0.51 (FES-ON). SMG-based RMSE ranged from 0.10 to 0.25 across all conditions and methods. Linear regression analysis produced $R^2$ values between 0.31 and 0.81 depending on joint, condition, and method. No significant RMSE difference was found between FES-ON and FES-OFF conditions within SMG. SMG RMSE values were also comparable to previously reported values (10-25%) in prior literature.

**Conclusion:**

Our findings suggest that sonomyography can be used as a noninvasive method for estimating joint kinematics when the joint movement is driven either by volition or by functional electrical stimulation. This technique can potentially be be useful in evaluating altered muscle dynamics and driving assistive and rehabilitation devices in individuals with neuromotor disorders such as cerebral palsy.

## Background

Functional limb movement occurs through a complex sequence of events that begins when the brain sends a signal through the central nervous system, passing through subcortical brain structures and the spinal cord before reaching the peripheral nervous system. This process results in the coordinated activation and contraction of specific muscles. Additionally, functional movement depends on sensory feedback from muscles and skin, which is transmitted back to the brain via the spinal cord, allowing the brain to adjust and refine movement. In individuals with neuromotor disorders, the typical flow of information through the motor signal pathway is disrupted at various levels, resulting in impaired movement [1]. Clinical assessments typically focus on the output of this signal chain, such as kinematic patterns or force production. However, specific mechanisms of impairment can vary across different points in the signal chain, depending on factors like injury severity and location, time elapsed since its occurrence, and rehabilitation interventions undertaken. Therefore, simultaneously quantifying multi-level impairments to develop a system-level characterization of the neuromuscular system represents a significant step toward a more accurate and comprehensive description of neuromotor dysfunction. This work demonstrates the proof of concept of such an approach, which requires a multimodal signal acquisition and time synchronization system that integrates multiple hardware and software components functioning cohesively.

Various technologies have been employed to assess the impact of neuromotor disorders on individuals. Surface electromyography (EMG) has been deployed to capture muscle activity and investigate the effect of neuromotor disorders on muscle activation patterns and intensity. EMG is being increasingly used as a tool to quantify and decode muscle activation [2], finding applications particularly in providing insights into muscular recruitment patterns [3], fatigue levels [4], and detecting co-contraction [5], all of which are crucial for quantifying rehabilitation effectiveness. Alongside being used to study the impact of neuromotor disorders, surface EMG signals have also been used to decode movement intent and consequently to control assistive devices used by individuals with neuromotor disorders. These approaches encompass pattern recognition [6], artificial neural networks [7], and regression-based methods [8, 9]. Despite its widespread adoption and dual use mentioned above, surface EMG has inherent limitations, including poor signal-to-noise ratios, random signal fluctuations [10], and low specificity between individual muscles due to cross-talk [11]. Furthermore, much of the advanced research on EMG control strategies has focused primarily on adult populations, healthy as well as those with stroke [12], amputation [13], and spinal cord injury [14]. Individuals with cerebral palsy (CP) present distinct additional challenges such as hyperreflexia, muscle weakness, increased antagonist co-contraction, and reduced force production [15, 16]. These unique characteristics complicate the use of EMG in this population, highlighting the need for tailored approaches to effectively deploy this technology. Electroencephalography (EEG) is another non-invasive technique with the capability to extract movement intent [17, 18]. While EMG and EEG are valuable tools for capturing the neural dynamics underlying movement, their utility is limited by several constraints. These technologies measure only the electrical activity associated with brain and muscle activation. However, many neuromotor disorders involve structural changes in the muscles themselves, which these methods cannot detect. Even though EMG can be used to infer many properties of the muscle, not all structural changes are captured. Hence, there is a critical unmet need for a non-invasive, reliable method to monitor muscle activity in individuals with neuromotor disorders, and integrate these data with brain and muscle electrical signals to develop a holistic, comprehensive model of muscle function in individuals affected with neuromotor disorders.

In this work, we have used CP as a test case, but this type of investigation would be beneficial for individuals with a range of neuromotor disorders. CP is the most common pediatric motor disorder affecting over 17 million people worldwide [19]. CP can affect one (unilateral) or both (bilateral) sides of the body, resulting in uncoordinated limb movements and decreased function [20, 21]. It presents with a large diversity of musculoskeletal impairments, which further compound with secondary effects of reduced physical activity, strength and endurance [22]. There are also alterations at the level of muscle structure in these disorders. Specifically, muscles in individuals with CP have been shown to be shorter and smaller in volume than those in typically developing children [23]. Additionally, muscles in these individuals have increased presence of connective tissue, which can contribute to muscle stiffness and spasticity [24]. These deficiencies in the muscles hamper movement performance, especially when combined with the effects that CP has on the other parts of the signal chain, from cortical activation to kinematics [25–27]. To address challenges in activities of daily living faced by individuals with CP, wearable devices such as robotic exoskeletons and functional electrical stimulation (FES) systems have been developed. These devices aim to either assist movement, train improved motor function, or both, helping to restore some degree of mobility and function [28]. To perform effectively, these devices must accurately and reliably detect movement intent and deliver appropriate torques or stimulation in real-time to support movement execution. A primary challenge in controlling wearable devices in individuals with movement disorders is that the relationship between neural drive, muscle dynamics, and movement is altered compared to individuals with typical development [29].

Most current work focuses on using kinematics from the user to decode motor intent [28]. Although this work has shown promise, kinematics may not always encode true movement intent due to motor impairments of users. Some exoskeletons have a hybrid system employing neuromuscular electrical stimulation (NMES) to achieve desired gait mechanics, for example, in children with crouch gait from CP [30], individuals with spinal cord injury [31] and healthy adults [2]. NMES is also called FES when it is used to elicit functional movement from an individual. Several studies have shown that NMES/FES can be used to strengthen muscles in clinical populations [32, 33], to increase muscle size or fiber diameter in individuals with CP [34, 35], and to reduce spasticity in survivors of stroke [36]. Although there has been significant work in using FES in combination with robotic or orthotic devices for therapeutic use [37, 38], this approach has its challenges. Ideally, stimulation parameters would adapt in real-time based on a number of factors such as movement intent, fatigue, nonlinear muscle recruitment, and movement variability. This is quite challenging to do using kinematics and/or EMG, due to the effect of spasticity as well as the presence of the FES signal in the EMG measurement. Although compensatory methods have been developed that filter out stimulation artifact from FES in the EMG signal [39–41], such a solution has not found its way into a clinical setting.

Motion capture systems provide highly accurate kinematic data but are generally confined to laboratory settings due to their size, cost, and lack of portability [42–44]. While motion capture systems can accurately measure kinematics, they are not portable and are often expensive to implement. In contrast, techniques like SMG offer a potentially more practical alternative as they are increasingly available as portable, wearable devices. Yet, a key gap is development and validation of algorithms to enable real-time kinematic decoding, especially when combined with assistive technology such as FES. The purpose of this work is a first step in filling this gap, by demonstrating feasibility of decoding movement, with the goal to continue development for eventual use in closed loop applications with assistive and/or rehabilitation devices.

EMG, on the other hand, is a wearable technology that has been extensively used for movement decoding and control. However, EMG signals are often noisy, especially in the presence of FES, and the signal patterns can vary significantly both across and within subgroups of clinical populations, partly due to altered neural drive associated with certain conditions. Ultrasound imaging (USI) presents a promising alternative, although it is not yet widely available for real-world applications. Recent advances in wearable USI systems [45–48] are bringing this technology closer to practical deployment. Unlike EMG, USI is a mechanical imaging method rather than an electrical sensing modality, and it can be used complementarily with EMG to improve the robustness and accuracy of motion decoding.

The emerging technique of Sonomyography (SMG), based on continuous USI of muscles during functional movement, offers a way to directly measure muscle deformation during dynamic movement, similar to electrical signals measured by EMG. SMG provides a non-invasive sensing modality that can spatially resolve individual muscles, including those deep inside the tissue [49–51]. Prior work has shown that SMG of forearm muscle can be used for real-time classification of multiple degrees of freedom in healthy individuals and individuals with transradial amputation [51], also allowing for proportional position control of a virtual cursor on a screen in able-bodied participants, prosthetic users, and persons with spinal cord injury [51–53]. Recent results [54, 55] have also shown the feasibility of using SMG as a control modality for exoskeletons [55, 56] and functional electrical stimulation [57, 58] alongside EMG in individuals with incomplete spinal cord injury or hemiplegia after stroke. As with other sensing modalities, SMG has limitations. A primary one is the size of the USI machine; most recent work has taken place with standard clinical systems. Additionally, the technique is sensitive to both probe placement and movement during measurement, which require attention during use to ensure proper contact. While it has been effective in able-bodied individuals as well as other clinical populations, it has not to date been examined in children and young adults with movement disorders such as CP. However, recent literature shows promise in these areas, as wearable USI technology continues to develop alongside clinical systems [45, 59].

USI has been extensively used to quantify structural changes in muscles affected by CP, providing valuable insights into muscle morphology and pathology, especially for early detection of CP [60, 61]. Clinically, USI is also employed to guide botulinum toxin injections and to assess muscle echogenicity using scales such as the Heckmatt scale [62–65]. Despite these applications, dynamic USI has not yet been widely used to directly infer joint kinematics. Our approach builds on methodologies developed in related fields, including work by other groups focusing on individuals with amputation [51], as well as studies in the population with spinal cord injury [53]. This growing body of research highlights the potential of SMG as a complementary technique to EMG for real-time motion decoding.

This study investigates the use of real-time SMG as a potential technique for detecting muscle deformation during movement, both with and without FES. It also demonstrates the proof of concept of simultaneous, time-synchronized multimodal sensing using EEG, EMG, SMG, and motion capture, which represents a novel approach to exploring the relationships between these signals, particularly in individuals with CP.

Specifically, we evaluated the accuracy of SMG in tracking limb kinematics under two conditions: voluntary movement (FES-OFF) and movement driven by FES (FES-ON). Participants performed single degree-of-freedom tasks spanning the range of motion (ROM) for two joints, the wrist and ankle, where extension movements were either voluntarily initiated or elicited via FES. We hypothesized that SMG would exhibit comparable tracking performance in both volitional and FES-driven movements.

**Fig. 1:**
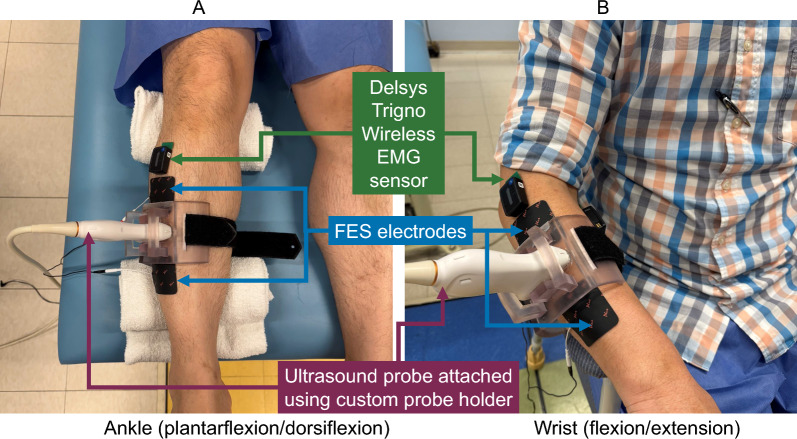
Sensor placement for multimodal data recording. Delsys Trigno Wireless EMG sensors were placed on the *extensor carpi radialis* (ECR) and *flexor carpi radialis* (FCR) during wrist flexion/extension, and the *medial grastrocnemius* (MG) and *tibialis anterior* (TA) during ankle plantarflexion/dorsiflexion. Samsung ultrasound LA2-14A probe was placed on the ECR during wrist flexion/extension, and slightly laterally to the TA during ankle dorsiflexion/plantarflexion. FES electrodes were placed on the TA and a more distal location for the ankle, and on the ECR and a more distal location for the wrist

**Fig. 2:**
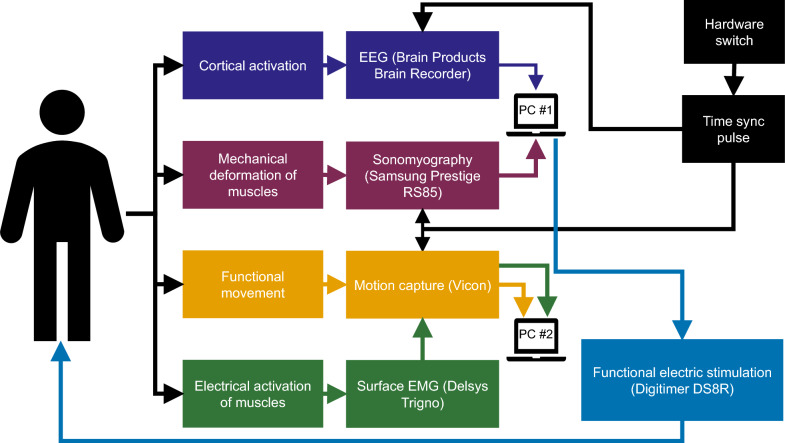
Schematic of the multimodal data collection setup. EEG and SMG data were recorded on PC#1, which also controlled stimulation frequency and amplitude. EMG and motion capture data were recorded on PC#2. Four modalities were recorded simultaneously to capture neural, muscular, anatomical, and kinematic information

## Methods

### Participants

We recruited 3 healthy volunteers and 1 individual with CP (GMFCS III, spastic diplegia), at the NIH Clinical Center (Table 1). The experiments in this study were approved by the National Institutes of Health Institutional Review Board under an observational clinical protocol (clinicaltrials.gov ID NCT06498596), with informed consent obtained prior to the experiments.

This study was designed as a technical feasibility study, with the primary objective of evaluating whether real-time USI can be effectively used to predict joint kinematics, and to test the integration and time synchronization of multiple data acquisition systems, including EEG, EMG, ultrasound, and motion capture. Because the study was not intended to generalize findings to the broader population, particularly individuals with CP, at this stage, no power analysis was conducted to determine sample size. Data collected and reported here will inform a more comprehensive future study with an appropriately powered sample.

### Experimental task

The participants performed single degree-of-freedom movements of the wrist (flexion/extension) and ankle (plantarflexion/dorsiflexion), with and without FES (Figs. 1 and 2). For the purposes of this study and to make it consistent with the wrist, we have defined dorsiflexion as ankle extension and plantarflexion as ankle flexion. EEG, surface EMG, USI, and motion capture were continuously recorded as movements were performed. These experiments were aimed at investigating the ability of USI to detect joint kinematics in the wrist and ankle for an individual with CP as well as healthy volunteers. Participants were seated on a chair for wrist movements, and in a semi-recumbent position on a bed for ankle movements. First, USI was recorded for 1 s on each joint during full flexion and full extension, to record the end states of the ROM. Then for each FES condition (ON or OFF), and each joint (wrist or ankle), full ROM movements were performed for 90 s. For the FES-OFF condition, a metronome played at 60 bpm and the participant was asked to complete movement to the full ROM in one direction at each beat, alternating between flexion/extension. During the FES-ON condition, the selected muscles (Table 2) were stimulated for 1-second intervals to drive wrist extension or ankle dorsiflexion, while wrist flexion or ankle plantarflexion were performed volitionally. The full time series of 90 s was used for analysis, with no data excluded based on time or ROM.

### Data collection and processing

We recorded multimodal signals at various points along the motor signaling chain, from brain activation to functional movement. Data were collected using EEG, EMG, USI, and motion capture. EEG was included as an adjunct measure for future analysis, while EMG and USI were assessed for their feasibility in extracting joint kinematics. Motion capture served as the ground truth for movement analysis.

#### Electroencephalography

EEG data were collected using a 64-channel Brain Products system (Brain Vision, Morrisville, NC, USA). The EEG data were streamed to PC#1 (AMD Ryzen Threadripper 3970X 32-Core Processor, 3.69 GHz, 64GB RAM, Windows 10), and recorded using BrainVision Recorder (version 1.27.0001) controlled in MATLAB (MathWorks, Inc., 2024A) via BrainVision’s Remote Control Server (RCS 2). Electrodes were configured according to the 10-20 international system (Easy Cap, Germany), and impedance was verified as below 20 kHz before data collection. EEGLAB open-source software [66] was utilized for EEG data processing.

The purpose of the EEG was as an adjunct, exploratory measure to assess (offline) cortical activity during FES-ON and FES-OFF conditions and its relationship to sEMG and SMG data.

**Fig. 3:**
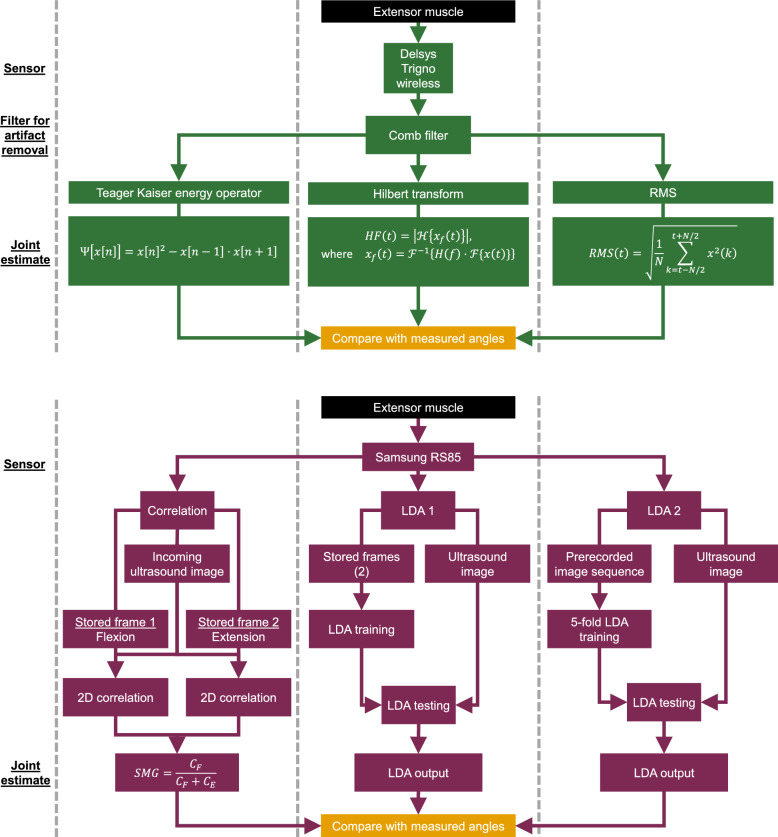
Schematic of the signal processing pipeline. EMG signals were recorded using a Delsys Trigno Wireless system and processed through a comb filter. Joint angles were then estimated using three methods: (1) Teager-Kaiser Energy Operator (TKEO), (2) Hilbert Transform, and (3) RMS Envelope. USI data was acquired using a Samsung Prestige RS85 system. Joint angles were estimated using three methods: (1) Correlation-Based Method, (2) Linear Discriminant Analysis (LDA) using end-state USI frames, and (3) LDA using time-series USI data

**Table 1:** Participant demographics

ID	CP diagnosis	Biological sex	Age	Side
P1	No	M	21 years, 9 months	R
P2	Yes	M	22 years, 0 months	L
P3	No	M	23 years, 10 months	R
P4	No	M	24 years, 5 months	R

**Table 2:** Stimulation parameters

	Wrist (Extensor carpi radialis)				Ankle (Tibialis anterior)			
Participant	Current high (mA)	Current low (mA)	Frequency (Hz)	Pulse width ($\mu$**s)**	Current high (mA)	Current low (mA)	Frequency (Hz)	Pulse width ($\mu$s)
P1	24	18	30	250	48	27	30	250
P2	20	15	30	250	23	19	30	250
P3	25	12	30	250	45	25	30	250
P4	20	10	35	250	33	15	35	250

The default starting values used were pulse width of 250 $\mu$s, frequency of 30 Hz, and stimulation amplitude of 10 mA

#### Surface electromyography

Muscle activation was recorded using a wireless surface EMG system (Trigno, Delsys, Boston, MA, USA). The EMG data were streamed to PC#2 (Intel(R) Xeon (R) W-2123 Processor, 3.6 GHz, 16GB RAM, Windows 10), and recorded by integrating with Vicon Nexus (version 2.17). The surface EMG sampling rate was 2000 Hz. Surface electrodes were attached to the *extensor carpi radialis* (ECR) and *flexor carpi radialis* (FCR) during wrist extension/flexion, and the *tibialis anterior* (TA) and *medial grastrocnemius* (MG) during ankle dorsiflexion/plantarflexion. Placement of surface EMG electrodes was according the SENIAM guidelines but sometimes placement had to be adjusted to account for the USI probe and FES electrodes.

To remove periodic artifacts arising from FES before applying feature extraction techniques, a comb filter was implemented with notch frequencies determined via power spectral analysis of the raw EMG recordings. EMG data were first band-pass filtered between 15 and 380 Hz, then passed through the Teager-Kaiser energy operator (TKEO) [67, 68], then low-pass filtered at 7 Hz (Fig. 3). The normalization of the EMG envelope was applied within each FES condition for each muscle and participant individually. Specifically, each 90-second EMG envelope recording was normalized to a zero to one scale. These normalized signals were then plotted against the measured motion completion level, which was normalized in the same way.

TKEO is a nonlinear signal processing technique used to estimate the instantaneous energy of a signal [67, 68]. For a discrete-time signal ($x\left[ n\right]$), the TKEO is computed using the formula:

1 $$ \Psi [ x [ n ]] =x [ n ] ^2-x [ n-1 ] \cdot x [ n+1 ] $$

This operator effectively captures the energy associated with rapid changes in the signal by combining the square of the current sample with the product of its neighboring samples. The TKEO is particularly useful for analyzing non-stationary signals such as EMG during dynamic muscle activation, as it enhances the detection of muscle activity while suppressing noise. Its computational simplicity and sensitivity to signal dynamics make it a popular choice for real-time signal processing applications.

Additionally, two other methods were used to predict joint kinematics from EMG. First, a Hilbert Transform was used to extract the EMG envelope. The raw EMG signal was first bandpass filtered between 20 and 450 Hz using a 4th-order Butterworth filter applied in zero phase via the *filtfilt* function in Matlab. The analytic signal was then computed using the Hilbert transform, and the EMG envelope was obtained as the magnitude (i.e., absolute value) of this complex analytic signal. This approach provides a smooth, continuous representation of muscle activation suitable for estimating joint kinematics. Additionally, an RMS filter was also separately applied to the EMG signal using a moving window size of 200 samples (0.1s at 2000Hz) with full overlap to smooth the signal and reduce high-frequency noise. This method helps highlight overall activation trends and was used to assess the feasibility of EMG-based joint angle estimation in this study.

#### Ultrasound imaging

Participants were instrumented with a clinical USI system (Samsung RS85) connected to a LA2-14A transducer, and used for B-mode imaging. The transducer was placed on the ECR during wrist extension/flexion, and slightly laterally to the TA during ankle dorsiflexion/plantarflexion. The imaging depth was set such that the bone remained within view during the full ROM for each joint. A USB-based video grabber (AV.io HDMI to USB 4K capture card, Epiphan Systems, Inc., ON, Canada) was used to transfer USI frame sequences in real-time to PC#1.

The USI probe was secured to the participant’s limb using a custom 3D-printed holder, fabricated on a Form 3+ printer (Formlabs, Sommerville, MA). This material provided a balance of strength and flexibility, making the probe holder rigid enough to securely hold the probe in place, yet flexible enough to conform initial probe insertion and varying limb diameters across participants. The probe holder was then fastened to the limb using a Velcro strap (Fig. 1). The USI probe weighs 144 gs, and the probe holder weighs 124 gs. The holder is fabricated from Flexible 80A Resin (FormLabs), a stiff yet soft-touch translucent elastomer with an 80A shore hardness that is tolerant to repeated bending, flexing, and compression. The combined weight of the probe and holder did not impede participant movement for any joint or condition in this study. None of the participants reported any discomfort related to the USI probe placement.

The acquired image frames were processed in MATLAB using custom-developed algorithms described in detail previously  [51, 69] and summarized here. First, training data were collected by recording 1 s of USI frames at fully flexed and fully extended states of each joint. Then, the USI frames were used to compute a signal that varied with the extent of muscle deformation. Defining $C_F$ as the 2-D correlation coefficient of the current frame to the average full flexion frame and $C_E$ as the 2-D correlation coefficient of the current frame to the average full extension frame, then the proportional signal (US) was computed as

2 $$\begin{aligned} US = \frac{C_F}{C_F+C_E} \end{aligned}$$

The 2D correlation coefficient (*C*) measures the similarity between two matrices (*A*) and (*B*) of size $m \times n$. It is computed by first subtracting the mean values $\bar{A}$ and $\bar{B}$ from each element of $A$ and $B$, respectively, to center the data. Then, the numerator calculates the sum of the element-wise products of these zero-mean matrices. The denominator normalizes this sum by the product of the square roots of the sums of squared deviations for each matrix. This normalization ensures that the coefficient (*C*) ranges between $-1$ and $1$, where $1$ indicates perfect positive correlation, $0$ indicates no correlation, and $-1$ indicates perfect negative correlation. The 2D correlation coefficient (*C*) was computed as

3 $$\begin{aligned} C = \frac{ \sum _{i=1}^{m} \sum _{j=1}^{n} (A_{ij} - \bar{A})(B_{ij} - \bar{B}) }{ \sqrt{ \sum _{i=1}^{m} \sum _{j=1}^{n} (A_{ij} - \bar{A})^2 } \cdot \sqrt{ \sum _{i=1}^{m} \sum _{j=1}^{n} (B_{ij} - \bar{B})^2 } } \end{aligned}$$

During the analysis, each USI frame was used to predict joint kinematics by comparing it to two reference frames obtained during the training phase, one corresponding to maximal flexion and the other to maximal extension. For each incoming USI frame, a proportional signal representing the joint angle was calculated by quantifying its similarity to these reference frames using a 2-D correlation metric (Eqs. 2, 3). This approach enabled continuous estimation of joint angles based on real-time USI data.

Our current approach predicts joint kinematics solely based on the current USI frame, following methodologies established in prior studies [51–53] involving other clinical populations. We did not incorporate algorithms that use information from previous USI frames to inform the current motion prediction. This choice was made to develop a position-based model that prioritizes high responsiveness to rapid muscle contractions initiated by the user. Although some existing methods [70] leverage temporal information from preceding frames to enhance motion prediction accuracy and signal smoothness, this often comes at the cost of decreased system responsiveness. In this study, we prioritized responsiveness by using frame-by-frame predictions, while recognizing that incorporating temporal dynamics may offer benefits worth exploring in future research.

Additionally, two approaches using Linear Discriminant Analysis (LDA) were implemented. In LDA Method 1, the model was trained using only two frames per trial (corresponding to full extension and full flexion), to evaluate estimation performance based on minimal training data in an analogous manner to the 2-D correlation SMG technique. In LDA Method 2, a 5-fold cross-validation approach was used, with 72 s of data (80%) from each trial used for training and the remaining 18 s (20%) for testing, allowing for a more generalized evaluation of performance across the full movement sequence.

#### Motion capture

A 12-camera motion capture system (Vicon Motion Systems Ltd, UK) recorded kinematic data at 100 Hz via reflective markers placed on the anatomical center of each joint and three non-collinear points on the adjacent limb segments to define the joint angles. The motion capture data were recorded in PC#2 using Vicon Nexus. A custom kinematic model was constructed in Visual3D software (C-Motion, Inc., Germantown, MD, USA) to track limb trajectory and compute joint angles. We created two separate models for tracking the ankle and wrist, each with its own dedicated set of markers. For the ankle tracking model, markers were placed on the medial and lateral malleoli to define the ankle joint center, and on the medial and lateral aspects of the knee joint using palpation to define the proximal reference. To define the foot segment, three non-collinear markers were placed on the first and fifth metatarsal heads and the heel. For the shank and thigh segments, four non-collinear markers were used on each segment to accurately capture their orientation. In the wrist tracking model, markers were placed on the medial and lateral wrist joint centers and on the medial and lateral elbow joints to define the distal and proximal reference points. Segment definition for the hand was achieved using three non-collinear markers placed on the hand, while a single marker was placed on the forearm to aid in segment tracking.

#### Functional electrical stimulation

We used a DS8R Biphasic Constant Current Stimulator (Digitimer Ltd., Welwyn Garden City, UK) to deliver stimulation current for inducing functional movement. The stimulation was controlled using PC#1, which sent commands to turn the stimulation on or off. This control was implemented via MATLAB running on PC#1, which communicated with an Arduino (programmed using Arduino IDE version 2.3.6) Teensy 3.2 microcontroller (PJRC, Sherwood, OR, USA) to manage the stimulation timing and delivery. During the FES-ON condition, electrical stimulation was alternated on and off in 1-second intervals. Prior to this, a separate calibration session was conducted to determine FES electrode placement and optimal stimulation parameters to achieve ankle dorsiflexion and wrist extension. Calibration began with an initial setting of 10 mA current amplitude, 250 $\mu$s pulse width, and 30 Hz frequency. The frequency was gradually increased to achieve a fused muscle contraction, and the current amplitude was increased until either functional movement through the full ROM was observed, or the participant reported the stimulation as uncomfortable. The stimulation settings determined during calibration were used throughout the FES-ON condition. This process was carried out for each participant (Table 2). We used ultrathin, flexible black foam-backed electrodes (ProMed Specialties, PA), either 2“$\times$4” or 2“$\times$2” in size, to deliver the stimulation.

Stimulation parameters were individually adjusted to maximize FES-driven movement within each participant’s comfort level. Although it is not clear whether these findings generalize to the larger population, the participant with CP had a lower tolerance threshold than the healthy volunteers, explaining the lower stimulation levels used. During calibration, stimulation was increased until either movement was elicited or discomfort reported. For the participant with CP, tolerance was reached before movement, unlike other participants. This study did not aim to assess effects of stimulation parameters on outcomes.

**Fig. 4:**
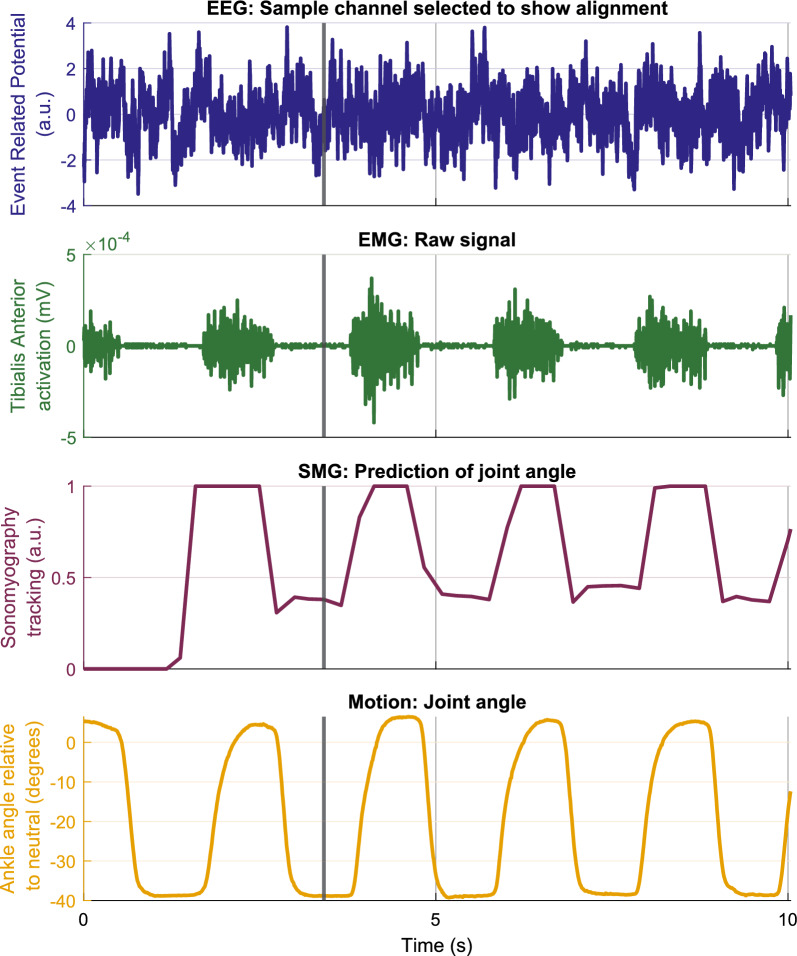
Sample time series from each recorded modality during FES-OFF condition from a healthy volunteer. Representative data from a single healthy participant (P4) while performing repeated ankle flexion/extension through their ROM. EEG, EMG, ultrasound imaging, and motion capture signals are shown to illustrate temporal dynamics and signal quality across the different data streams collected during the experimental task. A synchronization pulse (black vertical line) was used to align all the signals after acquisition, as shown in Fig. 2

**Fig. 5:**
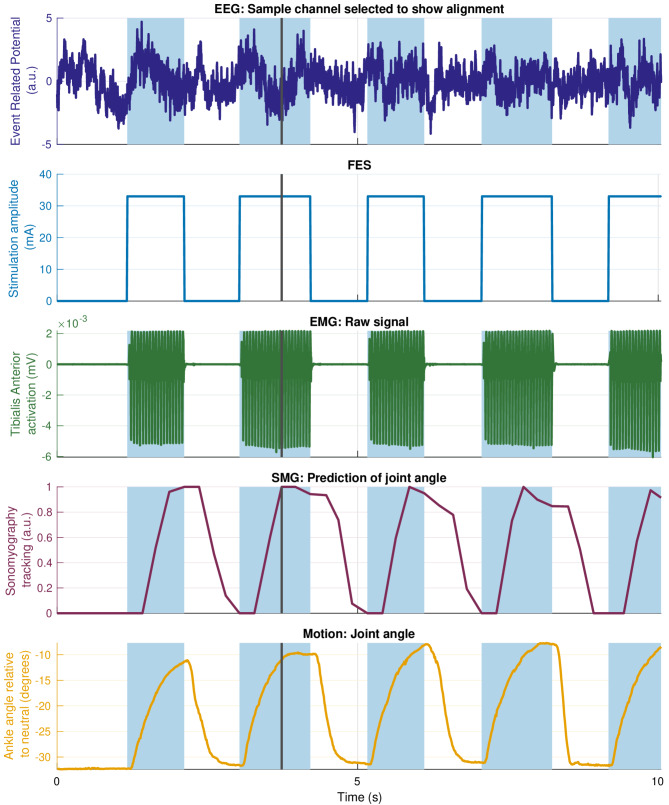
Sample time series from each recorded modality during FES-ON condition from a healthy volunteer. Representative data from a single healthy participant (P4) while performing repeated ankle flexion/extension through their ROM. EEG, EMG, ultrasound imaging, and motion capture signals are shown to illustrate temporal dynamics and signal quality across the different data streams collected during the experimental task. A synchronization pulse (black vertical line) was used to align all the signals after acquisition, as shown in Fig. 2. The blue shaded regions show when the FES was turned ON. It can be observed that while EMG data is adversely affected by FES artifacts, the SMG-based prediction of joint angles remains accurate

**Fig. 6:**
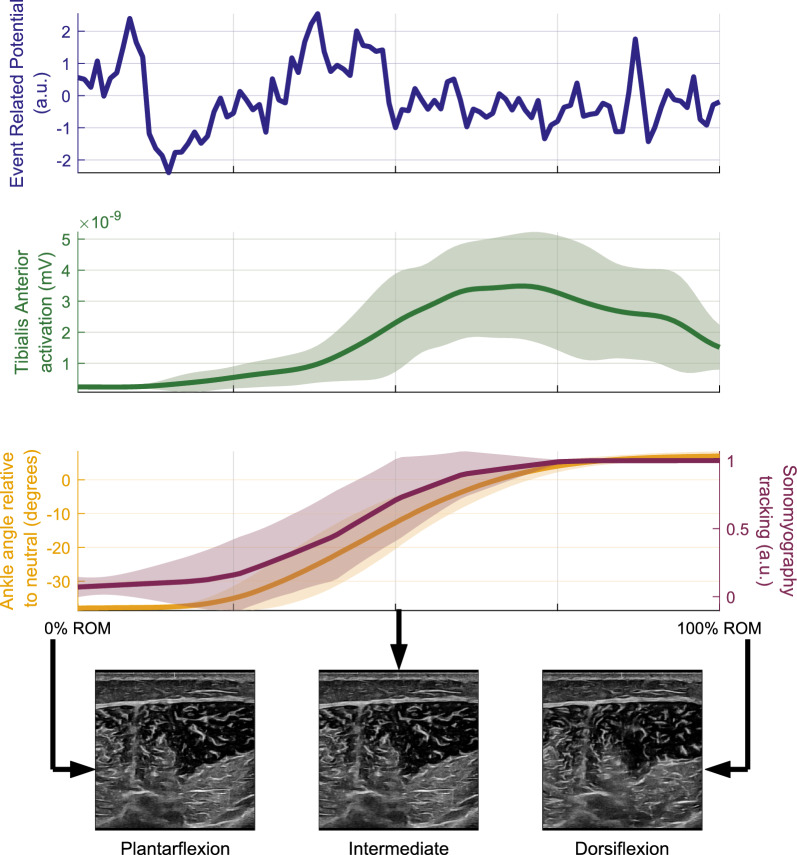
Representative data from a single healthy participant (P4) while performing repeated ankle flexion/extension through their ROM during the FES-OFF condition, with all movements superimposed. Example ultrasound images captured during the ROM movements are shown at the bottom

#### Data synchronization

To synchronize all data streams, we used a common 5V reference pulse. This pulse was delivered to the Vicon motion capture system. The Delsys data was also streamed to the Vicon system as an input, causing the motion capture and EMG data to be synchronized. The same 5V pulse was also sent to the Brain Products EEG system, and this pulse was marked as an event in the EEG file. For the USI system, the pulse was stepped down to 1.8V and fed to the USI machine via the ECGUSB1D-EX single-channel ECG add-on device which is meant to receive electrocardiogram signals. This signal appeared as a trace on the recorded USI frames. During post hoc analysis, we identified the first frame in which the pulse appeared on the USI frames, allowing us to align USI data with EEG, EMG, and motion capture recordings. This process ensured that EEG, EMG, ultrasound, and motion capture data could be synchronized offline with sufficiently low latency, such that the pulse was recorded in each stream within one sample of its arrival in the first data stream.

Two synchronization pulses were sent to each device. Let $P_1$ and $P_2$ represent the first and second pulse, respectively. The time points at which these pulses were recorded by each device are denoted as $P_1^{\textrm{EMG}}$,$P_1^{\textrm{EEG}}$,$P_1^{\textrm{US}}$, and similarly for $P_2$.

All data streams were aligned using the first pulse, setting

$$\begin{aligned} P_1^{\textrm{EMG}}=P_1^{\textrm{EEG}}=P_1^{\textrm{US}} \end{aligned}$$

This established a common reference point across all systems. To evaluate synchronization accuracy, we calculated the delay of the second pulse in each stream relative to the its time point in the EMG as a reference:

$$\begin{aligned} \Delta ^{\textrm{EMG}}&=P_2^{\textrm{EMG}}-P_2^{\textrm{EMG}}=0,\\ \Delta ^{\textrm{EEG}}&=P_2^{\textrm{EEG}}-P_2^{\textrm{EMG}},\\ \Delta ^{\textrm{US}}&=P_2^{\textrm{US}}-P_2^{\textrm{EMG}} \end{aligned}$$

Both the EEG and US recordings showed the second pulse arriving at the first sample recorded after it appeared in the EMG stream. This means the maximum synchronization error is identical to each system’s respective sampling rate. Specifically, EEG is 0.001s, for motion capture it is 0.01s, and for SMG it is $\approx$0.2s. Arbitrary units (a.u.) were used to represent normalized signal amplitudes in the figures wherever the signal was normalized as described earlier in this section.

### Sensor placement

Some adjustments were made to the standard SENIAM guidelines to accommodate the simultaneous placement of EMG sensors, USI probe, motion capture markers, and FES electrodes in close proximity. FES electrodes were first positioned on the muscle (one on belly, another at a distal location) to achieve effective joint extension. Subsequently, the USI probe and EMG sensors were placed to optimize EMG signal quality while ensuring the USI probe rested on the relevant region of the muscle. In instances where all devices could not be positioned on the same muscle site, the USI probe was shifted medially or laterally to enable the FES electrodes and EMG sensors to be placed correctly on their respective target locations. Although we measured flexors and extensors using EMG and USI, we primarily focused on the extensors muscles for extracting joint angle estimates (TA for ankle dorsiflexion and ECR for wrist extension).

### Outcome metrics and statistical analysis

Root Mean Squared Error (RMSE) was used to quantify the accuracy of joint angle predictions derived from SMG and EMG against ground truth joint angles obtained via motion capture. Given measured motion values $M = \{M_1, M_2, \ldots , M_N\}$ and predicted values $P = \{P_1, P_2, \ldots , P_N\}$, RMSE was calculated as:

$$\begin{aligned} \text {RMSE} = \sqrt{\frac{1}{N} \sum _{i=1}^N (M_i - P_i)^2} \end{aligned}$$

where $N$ is the number of samples.

To evaluate the linearity between predicted and actual kinematic signals, Pearson’s correlation coefficient $r$ was calculated for each joint and experimental condition. Statistical significance was assessed at $\alpha = 0.01$.

Comparisons of RMSE between experimental conditions (FES-ON vs. FES-OFF) were performed using the Wilcoxon signed-rank test, suitable for paired, non-normally distributed data. Differences in RMSE between modalities (EMG vs. SMG), which constitute independent groups, were assessed using the Mann–Whitney U test. Statistical significance was set at $\alpha = 0.01$ for all tests.

Effect sizes for non-parametric comparisons were calculated using the rank-biserial correlation coefficient $r$, appropriate for Wilcoxon signed-rank and Mann–Whitney U tests.

All statistical analyses were performed using MATLAB (version 2024A, The MathWorks, Natick, MA). Results are reported as mean ± standard deviation unless otherwise specified.

The primary hypotheses tested were as follows: (1) There is a statistically significant difference in RMSE between EMG and SMG modalities, reflecting differences in prediction accuracy; (2) There is a statistically significant difference in RMSE between FES-ON and FES-OFF conditions within each modality; and (3) The predicted joint angles from both EMG and SMG modalities exhibit a significant linear relationship with ground truth joint angles, as measured by Pearson’s correlation coefficient. For all tests, statistical significance was evaluated at the $\alpha = 0.01$ level.

## Results

We simultaneously collected multimodal data from EEG, EMG, USI, and motion capture, while the participants performed single degree-of-freedom movements driven whether by volition or FES. During the FES-OFF condition (Fig. 4), recorded EMG activity exhibited typical characteristic muscle activation patterns. In contrast, EMG signals recorded during the FES-ON condition saturated (Fig. 5). Nevertheless, the SMG signal modulated with kinematics without saturation under both conditions. The multimodal signals acquired using this system were time synchronized and used to study holistic neuromuscular function during a specific movement, for example, ankle dorsiflexion (Fig. 6). The sample results shown for ankle dorsiflexion demonstrate that EMG amplitude may not track kinematics, while SMG does track kinematics. This has been explored in much more detail in the next set of results.

**Fig. 7:**
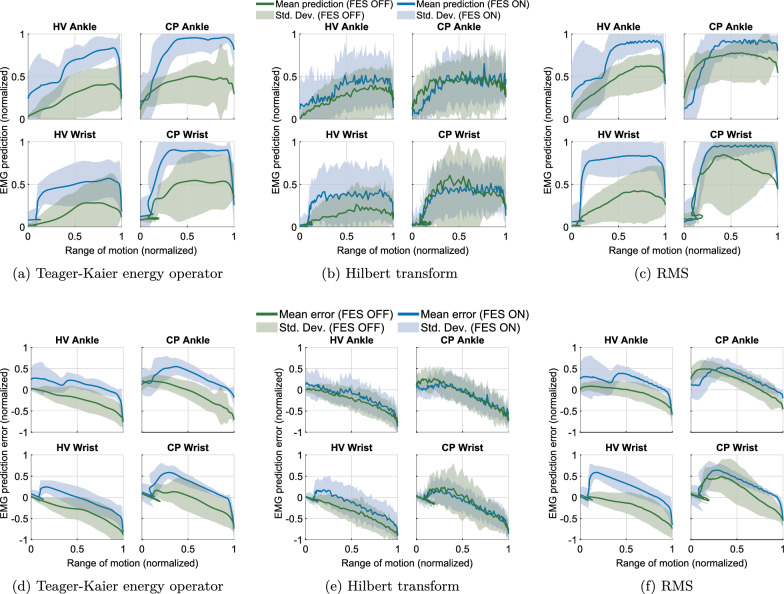
**a**–**c** Relationship between EMG-derived joint tracking and joint kinematics captured via motion tracking across the full ROM. Each EMG processing method is plotted separately: **a** Teager-Kaiser, **b** Hilbert Transform, and **c** RMS. Solid lines represent the mean error and the shaded region shows $\pm 1$ standard deviation. **d**–**f** Tracking error between EMG-predicted joint kinematics and motion capture data for the same dataset. Errors are shown separately for each EMG processing method: **d** Teager-Kaiser, **e** Hilbert Transform, and **f** RMS. Solid lines indicate mean error and shaded regions represent $\pm 1$ standard deviation. Each plot is normalized to its range

**Fig. 8:**
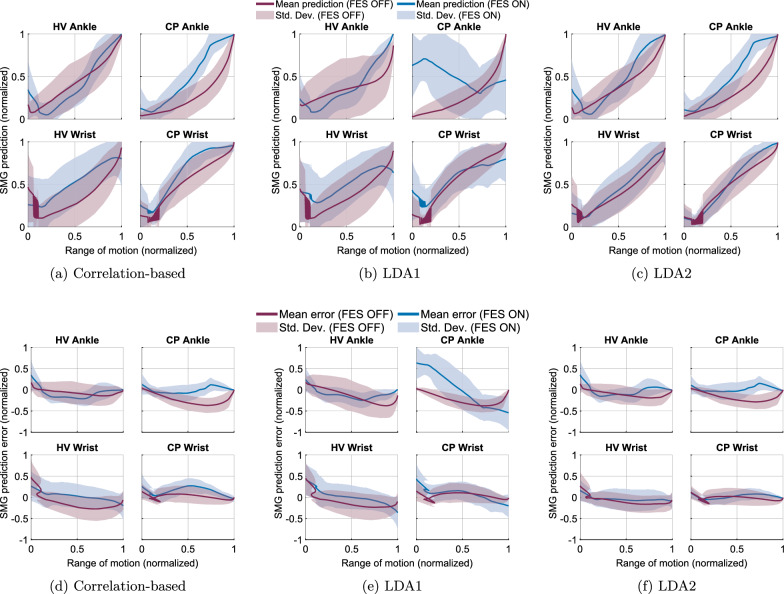
**a**–**c** Relationship between SMG-derived joint kinematics and joint kinematics captured via motion tracking. Each method is plotted separately: **a** Correlation-based, **b** LDA1, and **c** LDA2. Solid lines represent the mean and the shaded region shows $\pm 1$ standard deviation. **d**–**f** Tracking error between SMG-predicted joint kinematics and motion capture data for the same dataset. Errors are shown separately for each method: **d** Correlation-based, **e** LDA1, and **f** LDA2. Solid lines indicate mean error and shaded regions represent ±1 standard deviation. Each plot is normalized to its respective range

**Table 3:** Mean Tracking Errors for Healthy Volunteers and Individual with CP

Joint	FES-ON						FES-OFF					
	EMG			SMG			EMG			SMG		
	TK	HF	RMS	C	LDA1	LDA2	TK	HF	RMS	C	LDA1	LDA2
Healthy Volunteers												
Wrist	0.45 (0.39)	0.45 (0.39)	0.45 (0.39)	0.20 (0.22)	0.25 (0.26)	0.13 (0.15)	0.36 (0.39)	0.36 (0.39)	0.36 (0.39)	0.18 (0.22)	0.18 (0.21)	0.12 (0.16)
Ankle	0.51 (0.35)	0.51 (0.35)	0.51 (0.35)	0.14 (0.14)	0.14 (0.14)	0.12 (0.14)	0.48 (0.41)	0.48 (0.41)	0.48 (0.41)	0.12 (0.15)	0.25 (0.23)	0.12 (0.14)
Individual with CP												
Wrist	0.43 (0.34)	0.43 (0.33)	0.43 (0.33)	0.15 (0.14)	0.20 (0.19)	0.08 (0.07)	0.34 (0.33)	0.34 (0.33)	0.34 (0.33)	0.10 (0.10)	0.11 (0.11)	0.10 (0.09)
Ankle	0.48 (0.28)	0.48 (0.28)	0.48 (0.28)	0.48 (0.28)	0.48 (0.28)	0.12 (0.11)	0.57 (0.39)	0.57 (0.39)	0.57 (0.39)	0.17 (0.17)	0.18 (0.17)	0.13 (0.15)

*TK:* Teager-Kaiser, *HF:* Hilbert Transform, *RMS:* Root Mean Square, *C:* Correlation-based, LDA: Linear Discriminant Analysis. Mean values are shown with standard deviation in parentheses

### Prediction accuracy: Root Mean Squared Error (RMSE)

We evaluated tracking accuracy using RMSE between predicted joint angles (from EMG and SMG) and ground truth kinematics from motion capture (Table 3). Across all EMG-based methods (Teager-Kaiser, Hilbert Transform, and RMS), tracking errors were statistically significantly higher ($p<0.01$) compared to SMG-based predictions across all methods (Correlation method, LDA1 and LDA2). For SMG, because its frame rate was lower than that of the motion capture system, the error was calculated by comparing each SMG value to the closest corresponding value in the motion capture data based on timestamp. In contrast, for EMG, which had a higher frame rate than the motion capture system, the error was computed by comparing each motion capture measurement to the preceding EMG window.

For the EMG-based Teager-Kaiser method, RMSE values for healthy volunteers were 0.36 (±0.39) for the wrist and 0.48 (±0.41) for the ankle in the FES-OFF condition. With FES-ON, these values increased to 0.45 (±0.39) and 0.51 (±0.35), respectively. In the participant with CP, wrist RMSE was 0.34 (±0.33) with FES-OFF and 0.43 (±0.34) with FES-ON, while ankle RMSE decreased from 0.57 (±0.39) to 0.45 (±0.34) with stimulation. The Hilbert Transform and RMS methods showed similar results across all groups and joints.

In contrast, SMG-based methods resulted in lower RMSE values overall. For the correlation-based SMG method, healthy volunteer wrist RMSE was 0.18 (±0.22) without FES and 0.20 (±0.22) with FES. Ankle errors were 0.12 (±0.15) and 0.14 (±0.14), respectively. In the participant with CP, wrist RMSE was 0.10 (±0.10) without FES and 0.15 (±0.14) with FES, while ankle errors decreased from 0.17 (±0.17) to 0.11 (±0.11).

LDA-based SMG methods showed variability across versions. LDA1 produced wrist RMSEs of 0.18 (±0.21) and 0.25 (±0.26) for healthy volunteers (FES-OFF and FES-ON, respectively), and 0.11 (±0.11) and 0.20 (±0.19) for the participant with CP. Ankle RMSE for healthy volunteers dropped from 0.25 (±0.23) with FES-OFF to 0.14 (±0.14) with FES-ON. However, in the participant with CP, ankle errors increased from 0.18 (±0.17) to 0.48 (±0.28). The LDA2 method yielded the lowest SMG errors overall, with wrist RMSEs in healthy volunteers of 0.12 (±0.16) with FES-OFF and 0.13 (±0.15) with FES-ON. For the participant with CP, wrist RMSEs were 0.10 (±0.09) and 0.08 (±0.07), respectively.

### Correlation with ground truth

Linear regression analyses were performed separately for the wrist and ankle joints under FES-OFF and FES-ON conditions to assess the linearity between the predicted joint angles (from SMG/EMG) and the ground truth angles (Figs. 7, 8). For the wrist joint, the coefficient of determination R$^2$ was 0.5524 ($r = 0.74$) during volitional movement ($p<0.01$) and 0.52 ($r = 0.72$) during FES ($p<0.01$), indicating statistically significant linear relationships. Similarly, for the ankle joint, R$^2$ values were higher at 0.76 ($r = 0.87$) during FES-OFF ($p<0.01$) and 0.75 ($r = 0.87$) during FES-ON ($p<0.01$). These findings demonstrate significant linear correlations between predicted and actual joint kinematics across both experimental conditions.

For the LDA1 method, the wrist joint exhibited an R$^2$ of 0.56 ($r = 0.75$) during FES-OFF ($p<0.01$) and 0.31 ($r = 0.55$) during FES-ON ($p<0.01$), indicating statistically significant linear relationships. The ankle joint showed lower R$^2$ values of 0.49 ($r = 0.70$) during FES-OFF and 0.41 ($r = 0.64$) during FES-ON ($p<0.01$). LDA2 method also demonstrated linearity across all conditions, with wrist R$^2$ values of 0.75 ($r = 0.86$) during FES-OFF ($p<0.01$) and 0.76 ($r = 0.87$) during FES-ON ($p<0.01$), and ankle R$^2$ values of 0.80 ($r = 0.89$) during FES-OFF ($p<0.01$) and 0.78 ($r = 0.88$) during FES-ON ($p<0.01$).

A Mann–Whitney U test comparing the overall RMSE between SMG and EMG modalities showed a statistically significant difference ($p<0.01$), with SMG yielding lower tracking errors on average (effect size r = $-$0.93).

### FES-ON vs. OFF comparison

Across EMG methods, in healthy volunteers, FES-ON increased wrist RMSE from 0.36 to 0.45 and ankle RMSE from 0.48 to 0.51. In the participant with CP, FES-ON increased wrist RMSE from 0.34 to 0.43 but reduced ankle RMSE from 0.57 to 0.45. These patterns were consistent across the Teager-Kaiser, Hilbert, and RMS methods.

For SMG-based predictions, FES-ON had minimal or inconsistent effects. In healthy volunteers, wrist RMSE increased slightly (e.g., correlation-based method: 0.18–0.20), while ankle RMSE increased marginally or remained stable. In the participant with CP, FES-ON often led to increased wrist errors (e.g., LDA1: 0.11–0.20), while ankle RMSE varied by method—decreasing in the correlation-based method (0.17–0.11) but increasing in LDA1 (0.18–0.48).

Within the EMG modality, the comparison of RMSE values between FES-OFF and FES-ON conditions did not reach statistical significance ($p=0.624$, effect size $r = 0.125$). Similarly, within the SMG modality, no significant difference was observed between FES-OFF and FES-ON conditions ($p=0.507$, effect size $r = 0.167$).

### Comparison to Published Values

The SMG methods in this study demonstrated RMSE values that are comparable to previously reported SMG tracking errors of 10–25% (SD: 10–30%) from prior research [51–53]. Several configurations (e.g., LDA2) produced errors within or near the published SMG error range.

## Discussion

This work had two simultaneous goals. First, we investigated the technical feasibility of tracking volitional and FES-driven joint kinematics at the ankle and the wrist using SMG of the respective extensor muscles. The novelty of this manuscript lies in demonstrating for the first time the use of dynamic USI to decode kinematics across multiple joints in an individual with CP with and without FES. To test this, we tracked muscle deformation continuously as participants performed single degree-of-freedom movements of the wrist and ankle. The SMG signal linearly tracked joint kinematics for both joints (Fig. 8). Our results showed that SMG can estimate joint kinematics from muscle deformations during volitional and FES-driven movement. This technique has already shown to be useful in individual’s with amputation [51], and spinal cord injury [53]. To the best of our knowledge, we are the first to show its application in an individual with CP.

Our second goal was to investigate the technical feasibility of recording and time-synchronizing data streams from EEG, EMG, USI, and motion capture to facilitate future studies on motor control in CP. This study served as an initial technical feasibility assessment, with more comprehensive analyses planned using data collected during more complex tasks from a larger cohort of individuals with CP and their age- and sex-matched healthy controls.

### Synchronization of the data streams

Prior work [71] has demonstrated that EEG, EMG, and motion capture systems can be time-synchronized either through a shared triggering mechanism that initiates all systems simultaneously or by using an external voltage pulse for post hoc alignment. There are also other more complex setups proposed [72] to synchronize USI data with EMG, as well as combined ultrasound-EMG sensors [73]. In this study, we extend these approaches by incorporating continuous USI into the synchronized multimodal framework. We achieved this by sending a 5V reference pulse to the EEG, EMG, and motion capture systems for temporal alignment. To synchronize the USI data, the same pulse was routed through the ECG input of the USI machine, where it was plotted in real-time as an ECG trace on the screen. During post-processing, we used the first visible pulse in the USI recording as a temporal reference point, enabling accurate synchronization of the USI frames with the other physiological and kinematic data streams. We also used a secondary pulse that was initiated a few seconds after the first one to check the accuracy of time keeping in all the systems. If we aligned the first pulse, the second pulse should have also aligned in all the data streams. If this was not the case, it would mean that one data collection system has kept time at a rate different than the others. In our experimental setup, we found that this difference in time between the two pulses was less than the time it takes for one USI frame to be recorded and stored ($\approx 5$ frames per second).

### Stimulation parameters

The stimulation parameters we used in this experiment showed a high degree of variability from person to person (Table 2). We found that the individual with CP (P2) exhibited the lowest ’high’ FES threshold for the ankle, and one of the lowest ’high’ FES thresholds for wrist. P4 had the same ’high’ threshold for the wrist, but they had a higher frequency than P2. They also had a lower range between the minimum and maximum thresholds, compared to typically developing individuals. We set the higher threshold at the level that was either enough to elicit functional movement through the ROM, or if the participant was uncomfortable, whichever was lower of the two. For the individual with CP, we found that they reached their threshold for discomfort before the FES was high enough to induce movement through the full ROM. Although we did not investigate the exact reasons for decreased tolerance of FES in the participant with CP, contributing factors may include damage to the sensorimotor regions that alters sensory perception, altered muscle and nerve properties, changes in motor unit recruitment patterns, increased reflex activity, heightened startle response, changes in muscle fiber composition, and reduced muscle mass or subcutaneous fat [74–76].

Henneman’s size principle describes the orderly recruitment of motor units during voluntary muscle contractions, typically starting with smaller, fatigue-resistant units and progressing to larger, more fatigable ones as force demands increase. However, during functional electrical stimulation (FES), this natural recruitment order is often reversed, with larger motor units being activated first, or simultaneously, with smaller fibers. Consequently, the exact activation of motor units under external stimulation differ from those observed in voluntary contractions. This non-physiological activation often leads to rapid muscle fatigue and less controlled contraction [77, 78]. These effects may be further complicated in individuals with CP, whose muscle and neural properties can be substantially altered. It should be noted that this study does not make claims about population-level effects of FES-ON muscles in individuals with CP. Additionally, raw EMG recordings during FES require extensive filtering to remove stimulation artifacts, a process that can be challenging in practice. Prior studies [41] demonstrating effective artifact filtering often rely on simulated signals and assume fixed-frequency FES artifacts, conditions that are difficult to replicate with commercially available stimulators in real-world settings.

### Accuracy of tracking kinematics

Our results (Fig. 8) show that predictions of joint kinematics from SMG were accurate with low RMSE for the wrist (0.30) and ankle (0.21) in healthy volunteers and also for the individual with CP’s wrist (0.17) and ankle (0.20). However, the individual with CP had a much lower ROM at the ankle during the task, especially in the presence of FES. We used a step-wise increase in the stimulation parameters during calibration to fine tune it for each individual. For the individual with CP, especially for the ankle, the stimulation amplitudes were not sufficient to produce a strong enough level of muscle activation for joint movements through the ROM. This can be seen from the increased tracking error for the individual with CP from the ankle too. For the healthy volunteers, on average we could get functional movements through the ROM before the participant complained of discomfort. Further, we had three healthy volunteers but only one individual with CP. The purpose of this study was to establish the proof of concept of recording multimodal data from EEG, EMG, USI and motion capture, and also drive FES stimulation. Future work will focus on recruiting additional participants, and investigate similar techniques in other joints as well as investigate closed-loop control of FES stimulation.

On average, the kinematic tracking error using SMG was higher for FES-OFF, across both joint motions. This trend was consistent in both healthy participants and the individual with CP. One possible explanation is that the training data used to define maximum flexion and extension states were collected during volitional movement before each trial. Since the proportional signal predicted by SMG reflects muscle deformation, it may be influenced by the rate and pattern of that deformation. These factors are known to be affected by FES, which activates muscle fibers in a reverse recruitment order compared to natural movement [79, 80]. As a result, this may contribute to the slightly increased tracking error.

Adding more data, especially from the full ROM, improves the performance of the LDA estimator (Fig. 8). However, we chose to use training data from only two end-states during volitional movement for two main reasons. First, in real-world settings, training data can be updated regularly and easily collected by simply performing volitional ROM movements. Second, our results demonstrate that training data collected during volitional ROM alone is sufficient to accurately decode movement during FES-driven activity for both the LDA and correlation-based algorithms. Our goal was to minimize the amount of training data required while still maintaining effective decoding. To address this, we have included additional LDA results incorporating more training data. One benefit that LDA2 offers is that it had lower errors specifically for ankle tracking in the individual with CP, since it took the time series data for prediction and did not solely rely on the defined end-state USI frames (Fig. 8b vs Fig. 8c). Although previous studies have shown that just two frames of USI frames can enable robust decoding of joint kinematics in healthy individuals [52], persons with amputations [51], and individuals with spinal cord injury [53], we intend to expand our future analysis with more complex classifiers, especially for use in real-time applications.

### Data processing speeds

In the context of our study, the observed SMG frame rate during data recording was approximately 5 frames per second (fps), primarily due to limitations imposed by the current data handling approach. The USI system itself is capable of imaging at nearly 100 fps, depending on the chosen imaging settings. During data collection, USI frames were streamed in real time to a PC running MATLAB, where each frame was appended to a growing three-dimensional matrix for later post-processing. While streaming and initial processing could be performed at around 60 fps, the continuous appending of frames significantly reduced the effective frame rate to approximately 5 fps.

It is important to emphasize that in a real-time application, this data storage step would be eliminated, and MATLAB would not be used. This setup was selected for the current feasibility study to validate the concept. In a finalized, deployable system, particularly one employing wearable ultrasound, imaging would occur at approximately 50 to 60 fps with the decoding algorithm running on an embedded controller to enable responsive, real-time control. Previous studies [51–53, 81, 82] have similarly demonstrated that real-time use of clinical USI systems is feasible and effective for controlling prosthetic limbs and virtual interfaces. These applications have been validated in able-bodied individuals, prosthesis users, and individuals with spinal cord injuries, further supporting the practicality of real-time SMG-based control.

### Removal of FES-induced artifacts

We implemented an artifact suppression technique to address contamination in the EMG signals during FES. Specifically, we used power spectrum analysis to identify the relevant frequencies introduced by the stimulation and then implemented a comb filter targeting harmonics of the stimulation frequency. Several other filters haven been described in the literature [41, 83] as well.

While these filters offer partial improvements, one possible explanation for their limited effectiveness is variability in the stimulation frequency, which resulted from the MATLAB-Arduino control interface operating without closed-loop monitoring of the actual stimulation current. As a result, the stimulation frequency was not strictly fixed, leading to shifts in the harmonic components over time. Additionally, the EMG signal may be clipped by the Delsys Trigno Wireless System during the FES-ON condition.

These challenges reflect a common limitation when working with commercial, non-research-grade stimulators and underscores a broader issue in applying EMG for kinematic estimation during FES. Much of the existing literature relies on simulated EMG signals combined with idealized, fixed-frequency stimulation, which does not reflect the variability encountered in practical experimental settings. In our study, despite applying the most effective suppression technique prior to feature extraction using the Teager-Kaiser, Hilbert, and RMS filter, residual artifacts were still present in the EMG signal.

These findings illustrate the challenges of obtaining clean EMG signals during FES, particularly when stimulation frequency varies due to the absence of closed-loop control. As a result, even after applying multiple artifact suppression techniques, residual artifacts remained, which may affect the accuracy of EMG-based kinematic predictions. In contrast, SMG signals were less affected by stimulation-related artifacts in our setup, suggesting that SMG may offer a useful alternative for kinematic estimation under similar conditions. However, we emphasize that these observations are based on a limited number of participants and a specific experimental setup. It is likely that with improved hardware, such as more precise stimulators or higher-quality EMG sensors with better shielding or artifact rejection capabilities, some of these issues could be mitigated. Therefore, while SMG shows promise in this context, further studies with larger sample sizes and more controlled stimulation systems are necessary to draw broader conclusions about the relative strengths of each modality.

### Investigating neuromuscular function

These findings enable us to investigate the relationships between cortical activation, muscle function, and functional outcomes such as kinematics, in both healthy individuals and those with neuromotor impairments. The system described here not only supports this investigation successfully, but also records cortical activations (EEG), muscle activations (EMG), and functional outcomes (motion capture), while time-synchronizing them with USI frames (Figs. 4 and 5). Our future work will focus on quantifying the relationships between the recorded corticomuscular signals to assess impairment in function at each of these levels. This may enable the development of more objective metrics for evaluating neuromuscular impairment, rather than relying solely on clinical scales. While clinical scales quantify functional impairment, they are not sensitive to the underlying multimodal drivers of that impairment. The system demonstrated here may enable precision diagnosis, and potentially precision rehabilitation in the future. Although an individual with CP was recruited for this initial technical feasibility study, the data presented here are not sufficient to support claims of SMG’s motion prediction accuracy in general across the entire population of individuals with CP.

### Applications

These findings present SMG as an alternative for tracking joint kinematics during volitional movement as well as when the movement is driven by FES. One major potential application of this technique could be hybrid exoskeletons. Hybrid exoskeletons aid users by providing robotic and FES assistance during gait. The exoskeleton needs to accurately decode movement intent to adequately deliver this assistance. This is done by either measuring the kinematics of the joint directly using sensors in the exoskeleton, or by inferring intent from surface EMG signals. Both of these approaches have limitations. The measured kinematics are susceptible to perturbations from the user due to muscle spasticity. The surface EMG signal detects the delivered FES signal alongside volitional intent, making it difficult to differentiate between them. In this scenario, SMG offers a potential benefit of decoding movement intent from muscle deformations as opposed to electrical activity or kinematics. The recruitment order of muscle fibers is altered during functional movement driven by FES compared to volitional movement. Although not the focus of this study, this difference in muscle deformation could also potentially be picked up by SMG. Overall, these results demonstrate the capability of continuous USI to extract joint kinematics in real-time, and in that sense are comparable to previously published work [52, 84–86]. Ultrasound-based methods have been gaining popularity recently [49, 53], and have been used in applications related to clinical populations including amputees and spinal cord injury. There has been a growing literature [45–47, 59] on using wearable USI systems to characterize muscle function and control assistive devices. These advancements, combined with the continued development of EEG, EMG, and motion tracking systems, may eventually eliminate the need for a traditional research lab setup to collect such data. One of the key advantages of SMG over traditional systems like motion capture, particularly in neurorehabilitation applications, is the portability of the innovative USI sensors. These sensors are lightweight, wearable, and can be easily attached to the body, making them ideal for real-world use.

### Limitations

We used a correlation metric to derive the proportional SMG signal that tracks kinematics. We additionally used LDA-based approaches to improve our estimates of joint kinematics. However, there is now significant literature [87–89] exploring the use of more sophisticated machine learning algorithms for this purpose. We aim to use more complex models for future analysis. Our future work will focus on adding some of these more complex decoding algorithms and on real-time closed-loop device control using the derived SMG signal. For FES, we used a simple ON/OFF FES controller, but aim to use proportional FES controllers in our future work to control finely graded joint movement. The main challenge in using FES in the participant with CP was the heightened sensitivity to the stimulation, making it harder to achieve solely FES-driven ROM movements. A more graded approach to deliver FES instead of a ON/OFF approach may alleviate some of these issues. Additionally, our imaging frame rate for this work could also be improved by using better data streaming algorithms and storing some of the data locally.

## Conclusions

This work showed that SMG can track ankle and wrist kinematics in healthy volunteers as well as an individual with CP with high accuracy. The novelty of this manuscript lies in demonstrating for the first time the synchronization of dynamic USI with EEG, EMG and motion capture, as well as the use of USI to decode kinematics across multiple joints in an individual with CP with and without FES. Additionally, we show that accurate kinematic prediction can be achieved using only two USI frames, one captured at each end state of the ROM. Importantly, the prediction performance remains consistent whether the movement is driven by FES or by volitional effort. Future work will focus on developing closed-loop control of FES using SMG, using it with other assistive/rehabilitation technologies such as robotic exoskeletons, and evaluating the relationship between cortical and peripheral muscle activation and muscle fiber dynamics during functional movement.

## Data Availability

The datasets used and/or analyzed during the current study are available from the corresponding author on reasonable request.

## Abbreviations

B-mode: Brightness mode
bpm: Beats per minute
CP: Cerebral palsy
ECR: Extensor carpi radialis
EMG: Electromyography
EEG: Electroencephalography
FCR: Flexor carpi radialis
FES: Functional electrical stimulation
MG: Medial gastrocnemius
NMES: Neuromuscular electrical stimulation
SMG: Sonomyography
TA: Tibialis anterior
USI: Ultrasound imaging
